# Eight Weeks of *Bifidobacterium lactis* BL-99 Supplementation Improves Lipid Metabolism and Sports Performance through Short-Chain Fatty Acids in Cross-Country Skiers: A Preliminary Study

**DOI:** 10.3390/nu15214554

**Published:** 2023-10-27

**Authors:** Tieying Li, Zihan Rui, Letian Mao, Yashan Chang, Jing Shao, Yue Chen, Qi Han, Xuemei Sui, Nan An, Haoqiu Li, Haotian Feng, Tao Jiang, Qirong Wang

**Affiliations:** 1 Sports Nutrition Center, National Institute of Sports Medicine, Beijing 100029, China; 2 Key Lab of Sports Nutrition, State General Administration of Sport of China, Beijing 100029, China; 3 National Testing & Research Center for Sports Nutrition, Ministry of Science and Technology of the People’s Republic of China, Beijing 100029, China; 4 College of Exercise Science, Beijing Sport University, Beijing 100084, China; 5 College of Exercise & Health Science, Xi’an Physical Education University, Xi’an 710068, China; 6 Department of Exercise Science, Arnold School of Public Health, University of South Carolina, Columbia, SC 29208, USA; 7 Inner Mongolia Yili Industrial Group Co., Ltd., Hohhot 010110, China; 8 Inner Mongolia Dairy Technology Research Institute Co., Ltd., Hohhot 010110, China

**Keywords:** *Bifidobacterium*, BL-99, short chain fatty acid, lipid metabolism, sports performance, cross-country skiers

## Abstract

(1) Background: Probiotics in the form of nutritional supplements are safe and potentially useful for strategic application among endurance athletes. *Bifidobacterium animalis lactis* BL-99 (BL-99) was isolated from the intestines of healthy Chinese infants. We combined plasma-targeted metabolomics and fecal metagenomics to explore the effect of 8 weeks of BL-99 supplementation on cross-country skiers’ metabolism and sports performance. (2) Methods: Sixteen national top-level male cross-country skiers were recruited and randomly divided into a placebo group (C) and a BL-99 group (E). The participants took the supplements four times/day (with each of three meals and at 21:00) consistently for 8 weeks. The experiment was conducted in a single-blind randomized fashion. The subject’s dietary intake and total daily energy consumption were recorded. Blood and stool samples were collected before and after the 8-week intervention, and body composition, muscle strength, blood biochemical parameters, plasma-targeted metabolomic data, and fecal metagenomic data were then analyzed. (3) Results: The following changes occurred after 8 weeks of BL-99 supplementation: (a) There was no significant difference in the average total daily energy consumption and body composition between the C and E groups. (b) The VO_2max_ and 60°/s and 180°/s knee joint extensor strength significantly increased in both the C and E groups. By the eighth week, the VO_2max_ and 60 s knee-joint extensor strength were significantly higher in the E group than in the C group. (c) The triglyceride levels significantly decreased in both the C and E groups. In addition, the LDL-C levels significantly decreased in the E group. (d) The abundance of *Bifidobacterium animalis* increased two-fold in the C group and forty-fold in the E group. (e) Plasma-targeted metabolomic analysis showed that, after eight weeks of BL-99 supplementation, the increases in DHA, adrenic acid, linoleic acid, and acetic acid and decreases in glycocholic acid and glycodeoxycholic acid in the E group were significantly higher than those in the C group. (f) Spearman correlation analysis showed that there was a significant positive correlation between *Bifidobacterium animalis*’ abundance and SCFAs, PUFAs, and bile acids. (g) There was a significant correlation between the most significantly regulated metabolites and indicators related to sports performance and lipid metabolism. (4) Conclusions: Eight weeks of BL-99 supplementation combined with training may help to improve lipid metabolism and sports performance by increasing the abundance of *Bifidobacterium*, which can promote the generation of short-chain fatty acids and unsaturated fatty acids, and inhibit the synthesis of bile acids.

## 1. Introduction

Probiotics are defined by the FAO (Food and Agriculture Organization of the United Nations) and the WHO (World Health Organization) as live microorganisms that, when administered in adequate amounts, confer a health benefit on the host [1]. The main beneficial effects of probiotics relate to gastrointestinal (GI) symptoms, energy metabolism, immunity, nutrient absorption, and the regulation of oxidative stress [2,3,4]. *Bifidobacterium* are commensal microorganisms of the human gastrointestinal tract that are generally regarded as safe bacteria and widely used in functional foods and medicine [5,6]. *Bifidobacterium*’s biochemical roles in the human body include inhibiting the growth of harmful bacteria, synthesizing essential vitamins, promoting the absorption of minerals, generating organic acids (such as acetic acid, propionic acid, butyric acid, and lactic acid), and stimulating the immune system [7]. Their regulation of lipid metabolism is considered valuable for general health. In one study, fifty-one metabolic syndrome patients were divided into a control group and probiotic group; the probiotic group consumed fermented milk with *Bifidobacterium lactis* HN019 for 45 days. Compared with the baseline values and values in the control group, the probiotic group showed significant decreases in body mass index (BMI), T-CHO, LDL, tumor necrosis factor-α (TNF-α), and interleukin-6 (IL-6) [8]. However, the mechanism by which *Bifidobacterium* regulate lipid metabolism remains unclear.

Probiotics are considered a safe strategy for optimizing the health, sports performance, and recovery of athletes, especially endurance athletes [9]. Research on the application of *Bifidobacterium* in sports has increased in recent years. Eleven weeks of supplementation with *Lactobacillus* and *Bifidobacterium* could reduce the frequency and severity of gastrointestinal symptoms during exercise training and competition, and improve or maintain healthy intestinal barrier function [10]. An intervention that combined *Bifidobacterium* OLP-01 (isolated from an elite Olympic athlete) with a six-week exercise training program significantly improved grip strength and fatigue-associated indices (lactate, ammonia, creatine kinase (CK), lactate dehydrogenase (LDH), and glycogen content) [11]. It was reported that a combination of *Streptococcus thermophilus* and *Bifidobacterium* attenuated the range-of-motion decrements occurring after muscle-damaging exercise [12]. Another study involving six weeks of *Bifidobacterium longum* 35,624 supplementations in female swimmers did not show any effects on exercise performance or immune function, but the regimen did appear to alter cognitive function [13]. The results of probiotic interventions have been varied, possibly due to the use of different probiotic strains or multiple strains, differences in the time and dose of supplementation, or the use of different athletic cohorts [10]. The role of *Bifidobacterium* in improving exercise performance needs further research and exploration. It is generally confirmed that *Bifidobacterium* plays an important regulatory role in lipid metabolism [14]. During exercise, the energy supply ratio of sugar and fat varies with the duration and intensity of exercise. During exercise, according to the difference of intensity and time, the energy supply ratio of glucose and lipid is different. Long-chain and medium-chain fatty acids can be important fuel for energy expenditure during long-term endurance exercise [15].

With the development of metagenomics and metabolomics, it provides us more possibilities to explore the effect of *Bifidobacterium* supplementation on lipid metabolism and exercise performance. Physiological and biochemical demands might be more crucial in intense endurance events due to factors such as the intensity and length of the events and the temperature, and probiotics could be an important tool for improving overall health, performance, and energy availability [16]. We aimed to examine the effects of 8 weeks of *Bifidobacterium* supplementation on cross-country skiers’ lipid metabolism and exercise performance and the relationship between them by metabolomics and metagenomics.

## 2. Materials and Methods

### 2.1. Participants and Group

Sixteen national top-level male cross-country skiing athletes were recruited from the Shanxi Provincial Winter Sports Management Center. The average age of all the participants was 19.4 ± 0.9 years old, and the average length of their professional athletic training careers was 7.6 ± 3.7 years. After the initial recruitment process, a baseline examination of the athletes was conducted. The exclusion criteria included a history of cerebrovascular disease, hypertension, diabetes, impaired liver/kidney function, dairy allergy, digestive tract disorders, cardiovascular diseases, and metabolic disorders. All the participants signed informed consent forms before taking part. The study was approved by the Ethics Committee of the National Institute of Sports Medicine (approval no.: 202106); International Clinical Registration Number: ChiCTR2300069187.

The 16 participants were randomly divided into a C group (control, *n* = 8) and E group (experiment, *n* = 8) using a randomization table generated in Microsoft Excel. One subject was unable to complete the follow-up measures due to injury, and fifteen subjects completed the trial. This was a single-blind randomized controlled trial. The baseline information of each group is shown in Table 1.

### 2.2. Probiotic Supplementation Program

The C group only received ordinary yogurt and the E group received the same yogurt with the addition of 1 × 10^9^ CFU of *Bifidobacterium animalis* subsp. *Lactis BL-99* (BL-99). The supplementary solution was administered four times per day, being taken with each of three meals and at 21:00 before going to sleep. The supplementation period lasted for 8 weeks. The participants in each group were asked not to consume other yogurts or yogurt-containing foods during this study. All participants did not use other nutritional supplements during the intervention. We did not change the daily dietary habits of the participants.

### 2.3. Energy Intake and Expenditure

#### 2.3.1. Dietary Records

All the participants maintained their usual dietary habits throughout the experiment, except for the intake of dairy products being prohibited for one week prior to the experiment. The dietary analysis was conducted by the weighted food records based on a 2-day food diary (2 random days per week). Fluid consumption and snack intake were also recorded on the same day. Daily nutritional intake was analyzed using the Dietary Analysis and Management System for Athletes (developed by the National Institute of Sports Medicine, Beijing, China).

#### 2.3.2. Training Load and Energy Expenditure during Training

The Firstbeat Sport Sensor and Bodyguard 2 (Firstbeat Technologies Oy, Jyväskylä, Finland) were employed to monitor training load and energy expenditure during training. Training impulse (TRIMP) is an indicator generated by the FirstBeat algorithm, used to quantify the training load accumulated during a session; this was calculated based on the athlete’s heart rate reserve and the exercise duration of the session. TRIMP takes into account the intensity of exercise as calculated using the heart rate (HR) reserve method and the duration of exercise.

### 2.4. Body Composition

Body composition was measured using an InBody 570 Bioelectric Impedance Analyzer (InBody Corp., Seoul, Republic of Korea) every Monday morning.

### 2.5. Maximal Oxygen Consumption (VO_2max_)

During the test, all the participants were equipped with a metabolic system (SCHILLER ERGO AT104, Schiller, Switzerland) and were subjected to an increasing intensity exercise load test on a treadmill. The experimental protocol is mainly referring to Gasparini’s [17] and has been modified appropriately based on our laboratory protocol. The participants warmed up for three minutes at a speed of 1 km/h with a slope of 0 on the treadmill, and then, the load was increased by 1 km/h in terms of the speed and 1 degree in terms of the slope, with each round of load intensity maintained for one minute. When the running speed reached 14 km/h and the slope reached 12.5%, the participants were required to sprint until exhausted. The test was automatically terminated once two of the following three criteria were met: A. their heart rate was more than 180 beats per minute; B. even with encouragement, the participant was unable to maintain the predetermined intensity; and C. VO_2max_ had been reached, and real-time VO_2_ began to decrease.

### 2.6. Isokinetic Muscle Strength Test

The isokinetic muscle strengths of the ankle joint and knee joint were quantified using an IsoMed 2000 Dynamometer (D. & R. Ferstl GmbH, Hemau, Germany). Before commencing the test, the participants completed 3 duplicate practice trials. The protocol included an ankle and knee joint flexion and extension ability test at angular velocities of 60°/s and 180°/s, with 5 repetitions.

### 2.7. Hematological–Biochemical Analysis

In related to the condition of blood collection, the fasting states blood tests were assigned on the 1st day morning of the meso-cycle, which in this case were on the Mondays of the 1st & 9th week. In addition, training sessions were proceeded on Mondays through Saturdays weekly, and each Sunday was a designated resting day regardless of the weekly training load. Thus, choosing Monday as the blood-collecting day allows us to avoid possible errors due to training-related acute physiological responses. Blood samples were collected into 4.5 mL coagulation-promoting tubes. The blood samples were centrifuged at 3500 R/min for 15 min. Serum samples were analyzed using a Mindray BS420 Biochemical Analyzer (Shenzhen Mindray Scientific Co., Ltd., Shenzhen, China) to detect albumin (ALB), globulin (GLOB), total cholesterol (T-CHO), triglycerides (TG), high-density lipoprotein (HDL-C), and low-density lipoprotein (LDL-C).

### 2.8. Fecal Metagenomic Analysis

(1) Extraction of microbiome DNA and metagenome library preparation

The samples were subjected to quality control according to the sample type and product requirements. Genomic DNA was randomly fragmented. The fragmented genomic DNA was selected according to a certain average size, subjected to end-repair, and then 3′ adenylated. Adaptors were then ligated to the ends of these 3′ adenylated fragments. The PCR system and program were configured and set up to amplify the product. The corresponding library quality control protocol was selected depending on the product requirements. Single-stranded PCR products were produced via denaturation. Single-stranded circular DNA molecules were produced and replicated via rolling cycle amplification, and a DNA nanoball (DNB), which contains multiple copies of DNA, was generated. DNBs of sufficient quality were then loaded into patterned nanoarrays using the high-intensity DNA nanochip technique and sequenced through combinatorial probe-anchor synthesis (cPAS).

(2) Bioinformatic Analysis

All of the raw data were trimmed, and the host-originating reads were removed (only for samples of host origin). High-quality reads were de novo assembled, and contigs less than 300 bp in length were discarded. Genes were predicted and redundant genes were removed with an identity and coverage cut-off of 95% and 90%, respectively. Significant differences in alpha diversity between the groups were determined using estimated marginal means analysis applied to a linear mixed model, built with alpha diversity as the response variable, the different groups and time points as the predictor variables, and subject number as a random variable. To generate the annotation information, the protein sequences of the genes were aligned against the functional database KEGG with an E value cutoff of 1 × 10^−5^. Differentially enriched KEGG pathways were identified.

### 2.9. Targeted Metabolomic Analysis of Blood

In related to the condition of blood collection, the fasting states blood tests were assigned on the 1st day morning of the meso-cycle, which in this case were on the Mondays of the 1st & 9th week. In addition, training sessions were proceeded on Mondays through Saturdays weekly, and each Sunday was a designated resting day regardless of the weekly training load. Thus, choosing Monday as the blood-collecting day allows us to avoid possible errors due to training-related acute physiological responses. Blood samples were collected into 1 mL centrifuge tubes containing EDTA for plasma. The analytical instrument for this experiment was an LC-MS QTRAP 6500+ (SCIEX). The samples were analyzed in both positive and negative ion modes using a spray voltage of 4.5 kV and a capillary temperature of 350 °C. The mass scanning range was set at 50–1500 *m*/*z*. The nitrogen sheath gas and nitrogen auxiliary gas were set at flow rates of 30 L/min and 10 L/min, respectively. The HPLC-MS system was run in binary gradient mode. The mobile phase consisted of (A) a 0.1% formic acid aqueous solution and (B) a mixed acetonitrile–isopropanol solution. The gradient was set as follows: 0–1 min (5% B), 1–5 min (5–30% B), 5–9 min (30–50% B), 9–12 min (50–78% B), 12–15 min (78–95% B), 15–16 min (95–100% B), 16–18 min (100% B), 18–18.1 min (100–5% B), and 18.1–20 min (5% B). The flow rate was set to 0.2 mL/min. The pooled QC sample was injected five times at the beginning to ensure system equilibrium, and then, it was injected every five samples during plasma sample detection to further monitor system stability. A Waters’ BEH C18 column (2.1 mm × 10 cm, 1.7 μm, Waters) was used for all the analyses. The scaling method used in the PCA analysis is pareto correction, and the transformation method used is the log correction. Two hundred RPT was performed to avoid model over-fitting, and the VIP of each metabolite was obtained. Since univariate analysis is the simplest and most commonly used method for analyzing differential metabolites between two groups, the univariate analyses were also performed with fold change (FC) analysis and T-test to obtain FC value and *p* value using the R package metaX [18], respectively. For differential metabolites, metabolic pathway enrichment analysis was carried out based on the KEGG database, and metabolic pathways with *p* < 0.05 were significantly enriched by differential metabolites.

### 2.10. Data Analysis

SPSS 23.0 statistical analysis software was used to analyze the conventional indices. The data are shown as the mean ± standard deviation (mean ± SD), with *p* < 0.05 as the significance level. The Shapiro–Wilk test was used to test the normality of the data, and the repeated measure analysis of variance was used. Two-way ANOVA (time and supplementation) were used to compare the differences between groups. If there was an interaction effect between the time point and the BL-99 supplementation, we continued with simple main effect analysis. If there was no interaction effect, we continued with main effect analysis. Significance was defined as *p* < 0.05. The data are presented as the mean ± SD. In addition, effect size estimates (Cohen’s d) were calculated to assess and categorize efficacy as small (d = 0.2), medium (d = 0.5), or large (d = 0.8) [19]. The correlations of (1) *Bifidobacterium animalis* with the most regulated metabolites and (2) the most regulated metabolites with lipid-metabolism-related indicators and sports performance were assessed using Spearman’s rank correlation coefficient.

## 3. Results

### 3.1. Diary Nutrition

No significant differences in total energy intake or macronutrient intake were observed in either the C or E group over 8 weeks (Table 2).

### 3.2. Training Load and Energy Expenditure during Training

There were no significant changes in TRIMP or energy expenditure during the training of the participants in either the C or E group over 8 weeks (Table 3).

### 3.3. Body Composition

There was no significant change in the body composition of the participants in either the C or E group over 8 weeks (Table 4).

### 3.4. Maximal Oxygen Consumption (VO_2max_)

As shown in Table 5, after 8 weeks of BL-99 supplementation, the VO_2max_ was significantly increased in both the C and E groups. The VO_2max_ in the E group was increased significantly more than that in the C group.

### 3.5. Isokinetic Muscle Strength Test

As shown in Table 6, after 8 weeks of BL-99 supplementation, the 180°/s knee joint extensor strength had increased in both the C and E groups. The 60°/s knee joint flexor and extensor strength in the E group were significantly increased. By the eighth week, compared to in the C group, the 60°/s knee joint extensor strength in the E group had significantly increased.

### 3.6. Hematological–Biochemical Profiling

As shown in Table 7, after 8 weeks of BL-99 supplementation, the TG in both the C and E groups had significantly decreased. In addition, in the E group, the ALB levels significantly decreased and the LDL-C level significantly decreased.

### 3.7. Fecal Metagenomics

**Figure 1:**
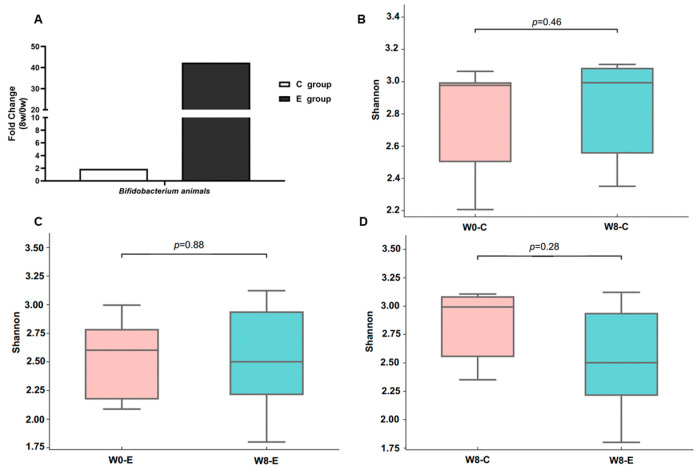
Overview of fecal metagenomics. (**A**) The fold change (8W/0W) in *Bifidobacterium animalis’* abundance in both the C and E groups after 8 weeks of BL-99 supplementation. (**B**) The alpha diversities difference of the C group after 8 weeks of BL-99 supplementation. (**C**) The alpha diversities difference of the E group after 8 weeks of BL-99 supplementation. (**D**) The alpha diversities difference between the C and E group after 8 weeks of BL-99 supplementation.

Figure 1A shows the fold change (8W/0W) in *Bifidobacterium animalis’* abundance in both the C and E groups after 8 weeks of BL-99 supplementation.

Figure 1B–D show the alpha diversities (Shannon indices) of the C and E groups after 8 weeks of BL-99 supplementation.

After 8 weeks of BL-99 supplementation, the *Bifidobacterium animalis* abundance in the intestinal flora had increased two-fold in the C group and forty-fold in the E group.

After 8 weeks of BL-99 supplementation, the alpha diversity (a measure of species diversity) of the gut microbiota (Shannon index) in both the C and E groups showed no significant difference. Meanwhile, there were no significant differences in the beta diversity of the gut microbiota or in the species abundance in each group.

Boxplot is a kind of statistical chart used to display the dispersion of a group of data, which arranges a group of data from large to small and calculates its upper edge (maximum), upper quartile Q3, median, lower quartile Q1 and lower edge (minimum), respectively. The boxplot represents the following (from bottom to top): ‘minimum’, first quartile (Q1), ‘median’, third quartile (Q3), and ‘maximum’. The test’s *p*-value is marked in the graph.

### 3.8. Plasma-Targeted Metabolomics

#### 3.8.1. Overview of Plasma-Targeted Metabolomic Analysis

**Figure 2:**
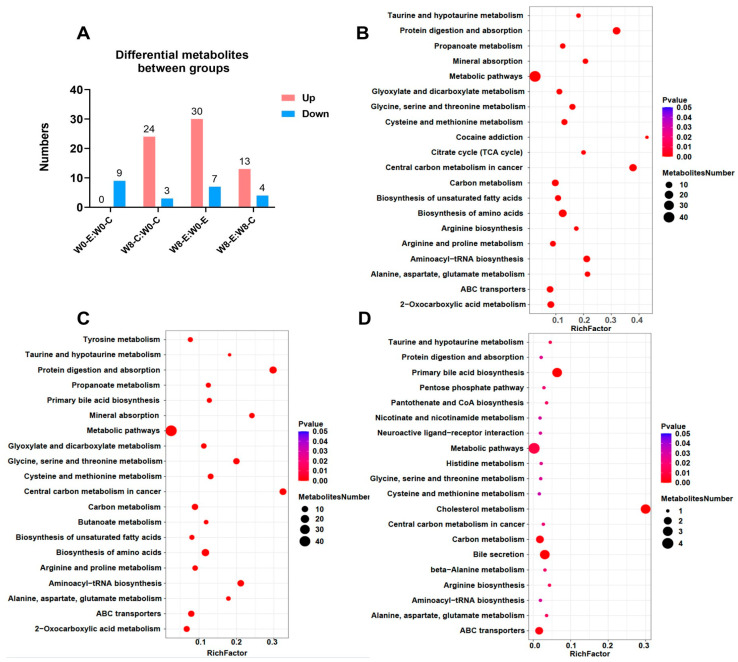
Overview of plasma-targeted metabolomic analysis. (**A**) Number of metabolites with concentrations that significantly differed between the groups. (**B**) Bubble plot of metabolic pathway enrichment analysis of differential metabolites in the C group after 8 weeks of BL-99 supplementation. (**C**) Bubble plot of metabolic pathway enrichment analysis of differential metabolites in the E group after 8 weeks of BL-99 supplementation. (**D**) Bubble plot of metabolic pathway enrichment analysis of differential metabolites between the C and E group after 8 weeks of BL-99 supplementation. The score graph of the PLS-DA analysis model of plasma-targeted metabolomic analysis both in the C and E groups after 8 weeks of BL-99 supplementation is shown in Supplementary Figure S1.

Figure 2A is the number of metabolites with concentrations that significantly differed between the groups.

Figure 2B shows the KEGG pathway analysis of differential metabolites in the C group after 8 weeks of BL-99 supplementation.

Figure 2C shows the KEGG pathway analysis of differential metabolites in the E group after 8 weeks of BL-99 supplementation.

Figure 2D shows the KEGG pathway analysis of differential metabolites between the C and E groups after 8 weeks of BL-99 supplementation.

As shown in Figure 2B,C, based on the KEGG database, after 8 weeks of BL-99 supplementation, in both the C and E groups, the most enriched pathways were the “Metabolic pathways” which included differential metabolites such as SCFAs, bile acids, and unsaturated fatty acids.

As shown in Figure 2D, after 8 weeks of BL-99 supplementation, when comparing the E group with the C group, the most enriched pathways were the “Metabolic pathways”, “primary bile acid biosynthesis”, etc.

Base on KEGG database, the metabolic pathway enrichment analysis of differential metabolites was carried out. The metabolic Pathway with p-value less than 0.05 was significantly enriched, and the bubble plot was drawn for the Pathway with significantly enriched differential metabolites. Enrichment factor on X-axis is the number of differential metabolites annotated to this Pathway divided by all identified metabolites annotated to this Pathway. The greater the value, the greater the proportion of differential metabolites annotated to this Pathway. The bubble size represents the number of differential metabolites annotated to the Pathway.

#### 3.8.2. The Most Regulated Lipid-Metabolism-Related Metabolites

**Figure 3:**
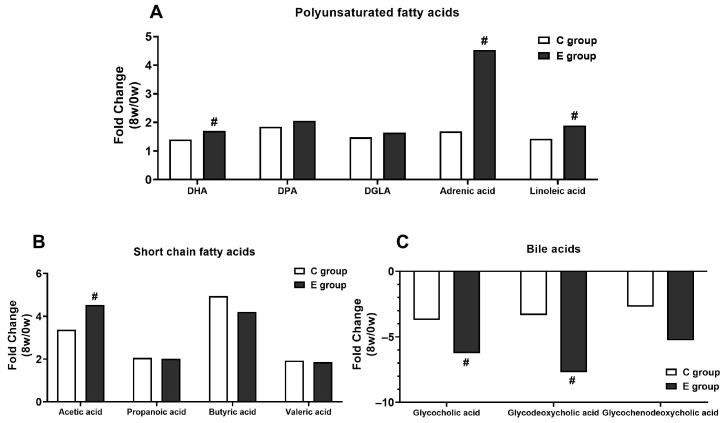
The most regulated lipid-metabolism-related metabolites. (**A**) The most regulated unsaturated fatty acids in both the C and E groups after 8 weeks of BL-99 supplementation. (**B**) The most regulated SCFAs in both the C and E groups after 8 weeks of BL-99 supplementation. (**C**) The most regulated bile acids in both the C and E groups after 8 weeks of BL-99 supplementation. # shows a significant difference between the C and E groups, #: *p* < 0.05.

Figure 3A–C show the fold changes (8 W/0 W) for the most regulated (*p* < 0.05) unsaturated fatty acids, SCFAs, and bile acids in both the C and E groups after 8 weeks of BL-99 supplementation. # indicates a significant difference between the C and E groups, #: *p* < 0.05.

Figure 3A The most regulated polyunsaturated fatty acids (PUFAs) including docosahexaenoic acid (DHA), docosapentaenoic acid (DPA), 8-11-14-eicosatrienoic acid (DGLA), adrenic acid, and linoleic acid. Compared to C group, the increase of DHA, adrenic acid and linoleic acid in the E group is significantly higher after 8 weeks of BL-99 supplementation.

Figure 3B The most regulated SCFAs including acetic acid, propanoic acid, butyric acid, and valeric acid. Compared to that in the C group, the increase in acetic acid in the E group was significantly higher after 8 weeks of BL-99 supplementation.

Figure 3C The most regulated bile acids including glycocholic acid, glycodeoxycholic acid, and glycochenodeoxycholic acid. Compared to that in the C group, the decrease in glycocholic acid and glycodeoxycholic acid in the E group was significantly higher after 8 weeks of BL-99 supplementation.

### 3.9. Correlation Analysis

#### 3.9.1. Correlation Analysis of *Bifidobacterium animalis* and the Most Regulated Metabolites

**Figure 4:**
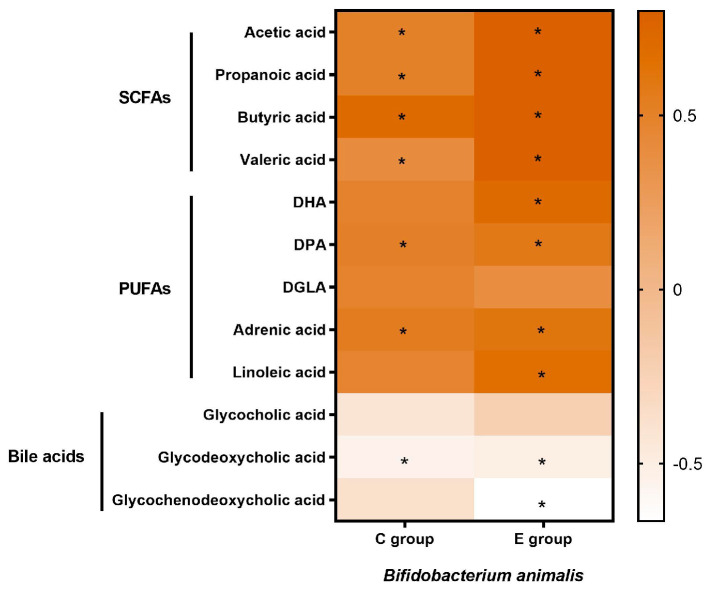
Correlation analysis of *Bifidobacterium animalis* and the most regulated metabolites. The color scale denotes the values of Spearman’s R from brown (positive correlation) to white (negative correlation). The significance levels according to the correlation tests are denoted as * *p* < 0.05.

As shown in Figure 4, Spearman correlation analysis shows that there are significant correlations between *Bifidobacterium animalis*’ abundance and SCFAs, PUFAs, and bile acids.

#### 3.9.2. Analysis of the Correlation of the Most Regulated Metabolites with Lipid-Metabolism-Related Indicators and Sports Performance

**Figure 5:**
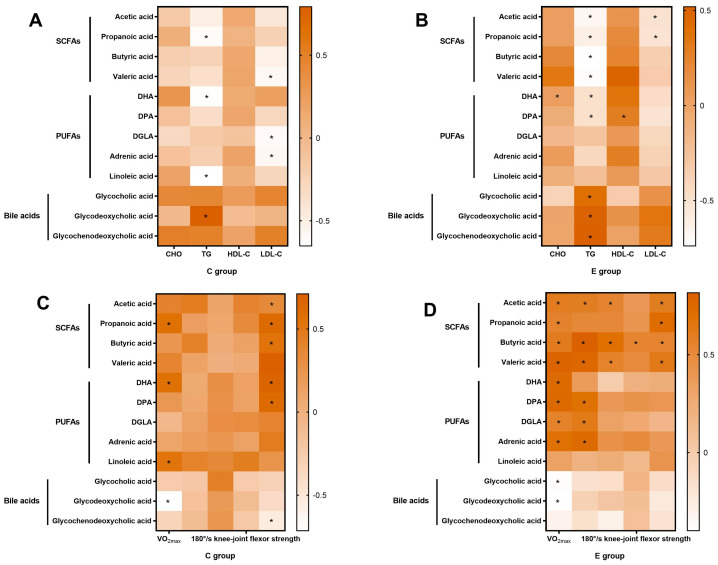
Analysis of the correlations of the most regulated metabolites with lipid-metabolism-related indicators and sports performance. (**A**) The Spearman correlations of the most regulated metabolites with lipid-metabolism-related indicators in the C group after 8 weeks of BL-99 supplementation. (**B**) The Spearman correlations of the most regulated metabolites with lipid-metabolism-related indicators in the E group after 8 weeks of BL-99 supplementation. (**C**) The Spearman correlations of the most regulated metabolites with sports performance in the C group after 8 weeks of BL-99 supplementation. (**D**) The Spearman correlations of the most regulated metabolites with sports performance in the E group after 8 weeks of BL-99 supplementation. The color scale denotes the values of Spearman’s R from brown (positive correlation) to white (negative correlation). The significance levels according to the correlation tests are denoted as * *p* < 0.05.

Figure 5A,B show the Spearman correlations of the most regulated metabolites with lipid-metabolism-related indicators in the C and E groups after 8 weeks of BL-99 supplementation.

Figure 5C,D show the Spearman correlations of the most regulated metabolites with sports performance in the C and E groups after 8 weeks of BL-99 supplementation.

## 4. Discussion

### 4.1. BL-99 Supplementation Increases Bifidobacterium Abundance

Several studies have demonstrated that supplementation with probiotics can increase the abundance of *Bifidobacteria* within the microbiota [7,20]. Multi-strain probiotic supplementation (5.0 × 10^9^ or 25 × 10^9^ CFU) for 4 consecutive weeks, followed by a 1-week washout, did not affect the composition of the subjects’ microbiota, but the abundance of *Bifidobacterium* in the feces of the high-dose group increased by 0.51 ± 0.26% [21]. In another study, a greater than 40-fold increase in the abundance of *Bifidobacterium animalis* was observed among 53 obese children who received 12-week treatment with supplementary probiotics [22]. *Bifidobacterium animalis* subsp. *lactis* BL-99 (BL-99) as a newly discovered probiotic in recent years, early research on BL-99 shows beneficial in modulating intestinal inflammation and function [23,24], and BL-99 also resulted in pronounced changes in the composition of the gut microbiota [24,25]. Our investigation demonstrated that following an 8-week period of BL-99 supplementation, the population of *Bifidobacterium animalis* increased two-fold within the C group and a remarkable fortyfold within the E group. These findings suggest that an 8-week regimen of BL-99 supplementation is effective in substantially elevating *Bifidobacterium* levels, while not significantly influencing gut microbiota diversity. The factors contributing to the increase in the abundance of certain bacterial strains while the overall composition of the gut microbiota remains relatively unchanged might stem from variations in the probiotic strains employed in previous studies, the wide spectrum of participants, variations in health conditions, and the consumption of diverse diets and nutrients by the volunteers. It’s crucial to recognize that the impact of probiotics is shaped not only by the specific strain but also by the complex interactions within the foundational microbial community [26]. In a study involving twenty new weight-loss individuals, all the participants received a low-carbohydrate, high-protein diet, and the symbiotic (treatment) group additionally received a synbiotic (probiotic plus prebiotic) supplement daily for 3 months. The researchers found that the supplement used in the study modified the relative abundance of gut bacteria and significantly increased the abundance of *Bifidobacterium*, by 200 times [27].

### 4.2. BL-99 Supplementation Combined with Training Ameliorated Lipid Metabolism through Short-Chain Fatty Acids

Both exercise and probiotic supplementation interventions exert an impact on lipid metabolism. A systematic review of the molecular networks involved in physical exercise stated that both chronic and acute bouts of exercise lead to significant changes in lipid metabolism [28]. After 24 weeks of a cardiovascular and resistance exercise intervention, 22 participants’ stool samples were collected, and the results showed that exercise was associated with significant increases in *Bifidobacterium* and butyrate [29]. It is evident from our results that, after an 8-week span of BL-99 supplementation combined with training, there were marked decreases in triglycerides, LDL-C, and bile acids, accompanied by substantial increases in SCFAs and polyunsaturated fatty acids (PUFAs) in both the C and E groups. This hints at the possibility that exercise in isolation could lead to improvements in athletes’ lipid metabolism.

The intervention of probiotic is another factor in improving lipid metabolism. The systematic review of 27 probiotic intervention studies also found that probiotics can ameliorate lipid profiles, SCFAs production, and the microbiota composition. Furthermore, the gut microbiota can affect lipid metabolism in the body by regulating the production of SCFAs, unsaturated fatty acids, and bile acids [30]. The incorporation of *Bifidobacterium animalis* (Probio-M8) into goat milk was shown to notably elevate the abundance of SCFAs and biologically active long-chain unsaturated fatty acids such as linoleic acid, a-linolenic acid, and docosahexaenoic acid [31]. Another investigation involving 134 participants were organized into 4 groups: (1) placebo, microcrystalline cellulose; (2) Litesse Ultra polydextrose (LU); (3) *Bifidobacterium animalis* subsp. *lactis* 420 (B420); and (4) LU + B420. Post a 6-month intervention, the plasma bile acids including glycocholic acid, glycoursodeoxycholic acid, taurohyodeoxycholic acid, and tauroursodeoxycholic acid were reduced in the LU + B420 group compared to the placebo group [32]. Exercise combined with probiotics can further improve body lipid metabolism. In a study conducted at the Institute of Cancer Research (ICR), mice were categorized into sedentary, exercise, *Bifidobacterium* OLP-01 (OLP-01), and exercise + OLP-01 groups. Following 6 weeks of intervention, the exercise + OLP-01 group exhibited significantly elevated levels of acetate, propionate, and butyrate levels compared to the sedentary and OLP-01 groups [11]. Our study findings underscore the significant differences between the E group and the C group, characterized by elevated DHA, adrenic acid, linoleic acid, and acetic acid levels, alongside reduced glycodeoxycholic acid and glycochenodeoxycholic acid concentrations. The evidence that polyunsaturated fatty acids (PUFAs) can enhance fat oxidation, and SCFAs can contribute to stabilizing blood glucose levels and fostering glycogen metabolism, suggests that incorporating BL-99 supplementation may enhance energy metabolism. This improvement could potentially lead to enhanced adaptability to training and improved athletic performance.

### 4.3. BL-99 Supplementation Combined with Training Increased Muscle Strength and VO_2max_ through Short-Chain Fatty Acids

Firstly, supplementation with probiotics has positive effects on aerobic metabolism, muscle strength, exercise endurance performance, and post-exercise muscle damage recovery [33,34]. Probiotics, prebiotics, SCFAs, and bacterial products are potential novel therapeutic agents for enhancing muscle mass and physical performance [35]. The potential mechanisms by which the microbiome modulates muscle mainly relate to cellular metabolism, inflammation, neuromuscular junctions, and mitochondrial function [35,36]. *Lactobacillus* and *Bifidobacterium* supplementation alleviates low-grade inflammation in the elderly by regulating the gut microbiota strains that ameliorate age-related muscle loss [37]. In a study examining 54 young healthy adults who took *Bifidobacterium* LP10 daily, an increase in muscle mass and dose-dependent increase in anti-fatigue capacity were observed [38]. Furthermore, *Bifidobacterium* may increase muscle strength by increasing SCFAs. Skeletal muscle is one of the target organs of SCFAs [39]. In one study, there was a significant difference in microbial composition between elite athletes and more sedentary controls; the elite athletes had higher levels of SCFAs than the controls [40]. Such a phenomenon may due to the athletic training routine; Barton and Peterson found that regular exercise can promote SCFAs production [40,41]. Therefore, the presence of high SCFAs levels stimulated by *Bifidobacterium* supplementation may further facilitate muscular synthesis. An animal study conducted by Lahiri et al. found that SCFA supplementation can increase the muscle mass (gastrocnemius muscle) and strength of mice [42]. This study’s outcomes revealed that among elite endurance athletes, BL-99 supplementation exerted a positive influence on the strength of both knee joint flexors and extensors in the E group. Remarkably, the E group displayed a significantly higher 60°/s knee joint extensor strength compared to the C group. Moreover, the correlation analysis revealed a substantial association between SCFAs and muscle strength within the E group, further support the notion that heightened SCFAs levels might serve as a key mechanism through which BL-99 supplementation contributes to muscle strength improvement.

There are few existent studies investigating the relationship between VO_2max_ and *Bifidobacterium* supplementation, yet several other studies have reported that supplementation with other probiotics increased the maximum oxygen uptake of the subjects. In a study that recruited sixty-six long-distance runners, a 12-week intervention with a probiotic supplement resulted in increased VO_2max_ for both male and female participants [43]. In another study, supplementation with *Saccharomyces boulardii* (Sb) increased the VO_2max_ of supplemented rats by 12.7% compared with controls (*p* = 0.01) [44]. In a study examining forty-six endurance swimmers supplemented with a probiotic yogurt or ordinary yogurt, a significant increase in VO_2max_ was observed in the probiotic yogurt group [45]. Probiotics can promote iron absorption and hemoglobin synthesis by regulating the balance of the gut microbiota, and SCFAs can directly promote hemoglobin synthesis [46,47,48]. There are also studies showing that VO_2max_ was unchanged among triathlon athletes after the ingestion of probiotics, but they still found significant improvements in SCFA levels and exercise endurance [49]. Our results showed that, after 8 weeks of *Bifidobacterium* supplementation, the VO_2max_ was significantly upregulated in both the C and E groups, and the correlation analysis showed a significant positive correlation between SCFAs and VO_2max_.

Studies have shown that a combination of sports training and probiotics has a better effect on sports performance than a single approach, such as exercise or probiotic supplementation alone. One study found that, compared with exercise or *Bifidobacterium* supplementation alone, exercise training combined with *Bifidobacterium* better ameliorated insulin sensitivity, blood glucose control, body composition, and physical performance [50]. In a study on marathon athletes supplemented with *Bifidobacterium* (10 × 10^9^ CFU/day) and *Lactobacillus* (10 × 10^9^ CFU/day) for 30 consecutive days, similar results were found: the combination of exercise and probiotics helped to maintain the total number of CD8 T cells and the immune reactions [51]. In another study, twenty-one subjects were divided into two groups: a placebo group and a *Bifidobacterium* OLP-01 (1.5 × 10^10^ (CFU)/day) group. The intervention lasted for five consecutive weeks and consisted of three weeks of regular training and two weeks of de-training. The results showed that OLP-01 significantly increased the change in the 12 min Cooper’s test running distance and the abundance of gut microbiota [52]. Although our study did not include a separate exercise-training-only group, the changes in multiple indicators in the E group were significantly higher than those in the C group; this also supports the theory that *Bifidobacterium* supplementation combined with training can improve sports performance.

To sum up, our preliminary and pilot study suggests that 8 weeks of BL-99 supplementation combined with training can improve lipid metabolism and exercise performance, and SCFAs may play an intermediary role in this process, but the mechanisms remain to be further explored.

## 5. Conclusions

Our study suggested that 8-week *Bifidobacterium lactis* BL-99 supplementation combined with training may help improve the lipid metabolism and sports performance of cross-country skiers by increasing the abundance of *Bifidobacterium*, which potentially promotes the generation of SCFAs and unsaturated fatty acids, and inhibits the synthesis of bile acids. In the future, coaches and athletes may consider use BL-99 to help improve the performance and lipid metabolism.

## Figures and Tables

**Table 1:** Baseline information of the participants.

Index	C Group (*n* = 8)	E Group (*n* = 7)
Age (years)	19.3 ± 0.7	19.6 ± 1.1
Training years	7.3 ± 0.3	7.5 ± 0.3
Height (cm)	178.6 ± 5.5	176.1 ± 3.7
Body mass (kg)	59.5 ± 8.4	59.9 ± 5.2
Fat mass (kg)	3.4 ± 1.3	3.5 ± 1.1
Muscle mass (kg)	31.8 ± 4.5	32.0 ± 1.2
Body Mass Index (BMI) (kg/m^2^)	18.6 ± 2.1	19.2 ± 1.1

**Table 2:** Nutrition according to diaries (mean ± SD).

Time	Index	C Group (*n* = 8)	E Group (*n* = 7)	*p* Value	Cohen’s d Effect Size
Week-0	Total energy (kJ)	3138.5 ± 304.6	3159.9 ± 196.3	0.63	−0.08
	Carbohydrate (g)	404.1 ± 56.1	402.9 ± 25.7	0.91	0.03
	Protein (g)	145.1 ± 8.8	148.4 ± 9.5	0.76	−0.36
	Fat (g)	106.8 ± 8.4	109.0 ± 8.4	0.38	−0.26
Week-8	Total energy (kJ)	3239.5 ± 915.2	3006.1 ± 622.8	0.36	0.30
	Carbohydrate (g)	373.5 ± 113.9	383.6 ± 76.9	0.16	−0.10
	Protein (g)	166.4 ± 38.4	148.6 ± 29.2	0.26	0.52
	Fat (g)	127.9 ± 44.1	106.6 ± 29.2	0.71	0.57

**Table 3:** Training load and energy expenditure (EE) during the training (mean ± SD).

Time	Index	C Group (*n* = 8)	E Group (*n* = 7)	*p* Value	Cohen’s d Effect Size
Week-1	TRIMP	126.6 ± 30.9	124.9 ± 21.3	0.71	0.06
	EE (Kcal)	2055.6 ± 306.5	2156.4 ± 389.8	0.73	−0.28
Week-2	TRIMP	207.3 ± 59.8	209.2 ± 55.8	0.35	−0.03
	EE (Kcal)	2697.9 ± 423.8	2778.6 ± 498.3	0.14	−0.17
Week-3	TRIMP	150.3 ± 46.2	175.4 ± 57.2	0.50	−0.48
	EE (Kcal)	2041.4 ± 299.1	2207.2 ± 352.7	0.07	−0.51
Week-4	TRIMP	168.5 ± 45.1	165.1 ± 46.3	0.61	0.07
	EE (Kcal)	2221.6 ± 392.7	2207.5 ± 406.4	0.15	0.04
Week-5	TRIMP	166.9 ± 38.0	161.0 ± 49.3	0.57	0.13
	EE (Kcal)	2062.5 ± 234.9	2135.8 ± 410.4	0.17	−0.22
Week-6	TRIMP	232.6 ± 73.1	232.1 ± 40.1	0.67	0.01
	EE (Kcal)	2399.4 ± 457.4	2458.4 ± 341.7	0.30	−0.15
Week-7	TRIMP	217.7 ± 31.3	214.0 ± 48.5	0.25	0.09
	EE (Kcal)	2455.0 ± 299.8	2212.4 ± 330.1	0.47	0.77
Week-8	TRIMP	134.5 ± 32.2	135.9 ± 43.4	0.20	−0.04
	EE (Kcal)	1872.4 ± 239.4	1562.2 ± 424.1	0.44	0.90

**Table 4:** The body composition of the participants (mean ± SD).

Time	Index	C Group (*n* = 8)	E Group (*n* = 7)	*p* Value	Cohen’s d Effect Size
Week-0	Body mass (kg)	59.5 ± 8.4	59.9 ± 5.2	0.93	−0.06
	Fat-free mass (kg)	56.2 ± 7.4	56.4 ± 5.0	0.94	−0.03
	Body fat (kg)	3.4 ± 1.3	3.5 ± 1.2	0.90	−0.08
	Body fat percentage (%)	5.6 ± 1.6	5.8 ± 2.0	0.82	−0.11
Week-8	Body mass (kg)	62.0 ± 8.0	62.1 ± 5.3	0.98	−0.01
	Fat-free mass (kg)	57.5 ± 7.0	57.6 ± 5.0	0.98	−0.02
	Body fat (kg)	4.4 ± 0.5	4.5 ± 0.4	0.98	−0.22
	Body fat percentage (%)	7.1 ± 1.9	7.2 ± 1.6	0.90	−0.06

**Table 5:** Maximal oxygen consumption (VO_2max_) (mean ± SD).

**Time**	**Index** **(mL/Kg)**	**C Group (*n* = 8)**	**E Group (*n* = 7)**	***p* Value**	**Cohen’s d** **Effect Size**
Week-0	VO_2max_	55.9 ± 4.4	55.8 ± 5.4	0.56	0.02
Week-8	VO_2max_	61.8 ± 3.2 *	64.5 ± 2.6 *#	0.02	−0.93
**Group**	**Index** **(mL/Kg)**	**Week-0**	**Week-8**	***p* Value**	**Cohen’s d** **Effect Size**
C group (*n* = 8)	VO_2max_	55.9 ± 4.4	61.8 ± 3.2 *	0.01	−1.53
E group (*n* = 7)	VO_2max_	55.8 ± 5.4	64.5 ± 2.6 *#	0.01	−2.05

* shows a significant difference in the same supplement group, * p < 0.05. # indicates a significant difference at the same time point, *# p* < 0.05.

**Table 6:** Isokinetic strength (peak torque to body weight ratio, PT/BW) (mean ± SD).

**Time**	**Index** **(Right + Left, Nm/Kg)**	**C Group (*n* = 8)**	**E Group (*n = 7*)**	***p* Value**	**Cohen’s d** **Effect Size**
Week-0	60°/s knee joint flexor strength	2.3 ± 0.4	2.0 ± 0.4	0.11	0.75
	60°/s knee joint extensor strength	5.2 ± 0.6	5.0 ± 0.4	0.55	0.39
	180°/s knee joint flexor strength	2.1 ± 0.2	2.2 ± 0.3	0.56	−0.39
	180°/s knee joint extensor strength	3.9 ± 0.4	4.0 ± 0.5	0.61	−0.22
Week-8	60°/s knee joint flexor strength	2.8 ± 0.5	2.9 ± 0.6 *	0.70	−0.18
	60°/s knee joint extensor strength	5.1 ± 0.2	5.7 ± 0.4 *#	0.00	−1.90
	180°/s knee joint flexor strength	2.3 ± 0.5	2.5 ± 0.6	0.59	−0.36
	180°/s knee joint extensor strength	4.5 ± 0.5 *	4.8 ± 0.6 *	0.24	−0.54
**Group**	**Index** **(Right + Left, Nm/Kg)**	**Week-0**	**Week-8**	***p* Value**	**Cohen’s d** **Effect Size**
C group (*n* = 8)	60°/s knee joint flexor strength	2.3 ± 0.4	2.8 ± 0.5	0.06	−1.10
	60°/s knee joint extensor strength	5.2 ± 0.6	5.1 ± 0.2	0.77	0.22
	180°/s knee joint flexor strength	2.1 ± 0.2	2.3 ± 0.5	0.24	−0.53
	180°/s knee joint extensor strength	3.9 ± 0.4	4.5 ± 0.5 *	0.02	−1.33
E group (*n* = 7)	60°/s knee joint flexor strength	2.0 ± 0.4	2.9 ± 0.6 *	0.00	−1.77
	60°/s knee joint extensor strength	5.0 ± 0.4	5.7 ± 0.4 *#	0.01	−1.75
	180°/s knee joint flexor strength	2.2 ± 0.3	2.5 ± 0.6	0.25	−0.63
	180°/s knee joint extensor strength	4.0 ± 0.5	4.8 ± 0.6 *	0.02	−1.45

* shows a significant difference in the same supplement group, * *p* < 0.05. # shows a significant difference at the same time point, # *p* < 0.05.

**Table 7:** Hematological–biochemical profiles of the participants (mean ± SD).

**Time**	**Index**	**C Group (*n* = 8)**	**E Group (*n* = 7)**	***p* Value**	**Cohen’s d** **Effect Size**
Week-0	GLOB (g/L)	28.9 ± 3.0	26.3 ± 1.4	0.77	1.11
	ALB (g/L)	47.2 ± 1.7	46.6 ± 1.4	0.48	0.38
	TG (mmol/L)	0.7 ± 0.3	0.6 ± 0.2	0.19	0.39
	T-CHO (mmol/L)	3.2 ± 0.4	3.4 ± 0.7	0.67	−0.35
	HDL-C (mmol/L)	1.4 ± 0.2	1.5 ± 0.2	0.55	−0.50
	LDL-C (mmol/L)	2.0 ± 0.5	2.1 ± 0.6	0.87	−0.18
Week-8	GLOB (g/L)	28.5 ± 1.9	25.4 ± 2.0	0.05	1.59
	ALB (g/L)	45.6 ± 1.5	44.8 ± 0.8 *	0.22	0.67
	TG (mmol/L)	0.3 ± 0.1 *	0.3 ± 0.1 *	0.52	0.00
	T-CHO (mmol/L)	3.3 ± 0.5	3.2 ± 0.5	0.67	0.20
	HDL-C (mmol/L)	1.5 ± 0.2	1.5 ± 0.2	0.77	0.00
	LDL-C (mmol/L)	1.6 ± 0.5	1.6 ± 0.4 *	0.91	0.00
**Group**	**Index**	**Week-0**	**Week-8**	***p* Value**	**Cohen’s d** **Effect Size**
C group (*n* = 8)	GLOB (g/L)	28.9 ± 3.0	28.5 ± 1.9	0.77	0.16
	ALB (g/L)	47.2 ± 1.7	45.6 ± 1.5	0.08	1.00
	TG (mmol/L)	0.7 ± 0.3	0.3 ± 0.1 *	0.00	1.79
	T-CHO (mmol/L)	3.2 ± 0.4	3.3 ± 0.5	0.75	−0.22
	HDL-C (mmol/L)	1.4 ± 0.2	1.5 ± 0.2	0.30	−0.50
	LDL-C (mmol/L)	2.0 ± 0.5	1.6 ± 0.5	0.11	−0.80
E group (*n* = 7)	GLOB (g/L)	26.3 ± 1.4	25.4 ± 2.0	0.44	0.52
	ALB (g/L)	46.6 ± 1.4	44.8 ± 0.8 *	0.01	1.58
	TG (mmol/L)	0.6 ± 0.2	0.3 ± 0.1 *	0.01	1.90
	T-CHO (mmol/L)	3.4 ± 0.7	3.2 ± 0.5	0.62	0.33
	HDL-C (mmol/L)	1.5 ± 0.2	1.5 ± 0.2	0.95	0.00
	LDL-C (mmol/L)	2.1 ± 0.6	1.6 ± 0.4 *	0.04	0.98

* shows a significant difference in the same supplement group, * p < 0.05.

## Data Availability

Data are available on request due to privacy and ethical restrictions.

## Supplementary Materials

The following supporting information can be downloaded at: https://www.mdpi.com/article/10.3390/nu15214554/s1, Figure S1: Score graph of the PLS-DA analysis model of plasma-targeted metabolomic analysis.
