# Supplementary material for: Eight Weeks of Bifidobacterium lactis BL-99 Supplementation Improves Lipid Metabolism and Sports Performance through Short-Chain Fatty Acids in Cross-Country Skiers: A Preliminary Study
Source: Nutrients. 2023 Oct 27;15(21):4554. doi: 10.3390/nu15214554 (PMC10648242; doi:10.3390/nu15214554)
Supplement: Supplementary file 1 [file nutrients-15-04554-s001.zip › nutrients-2616126-supplementary.pdf]

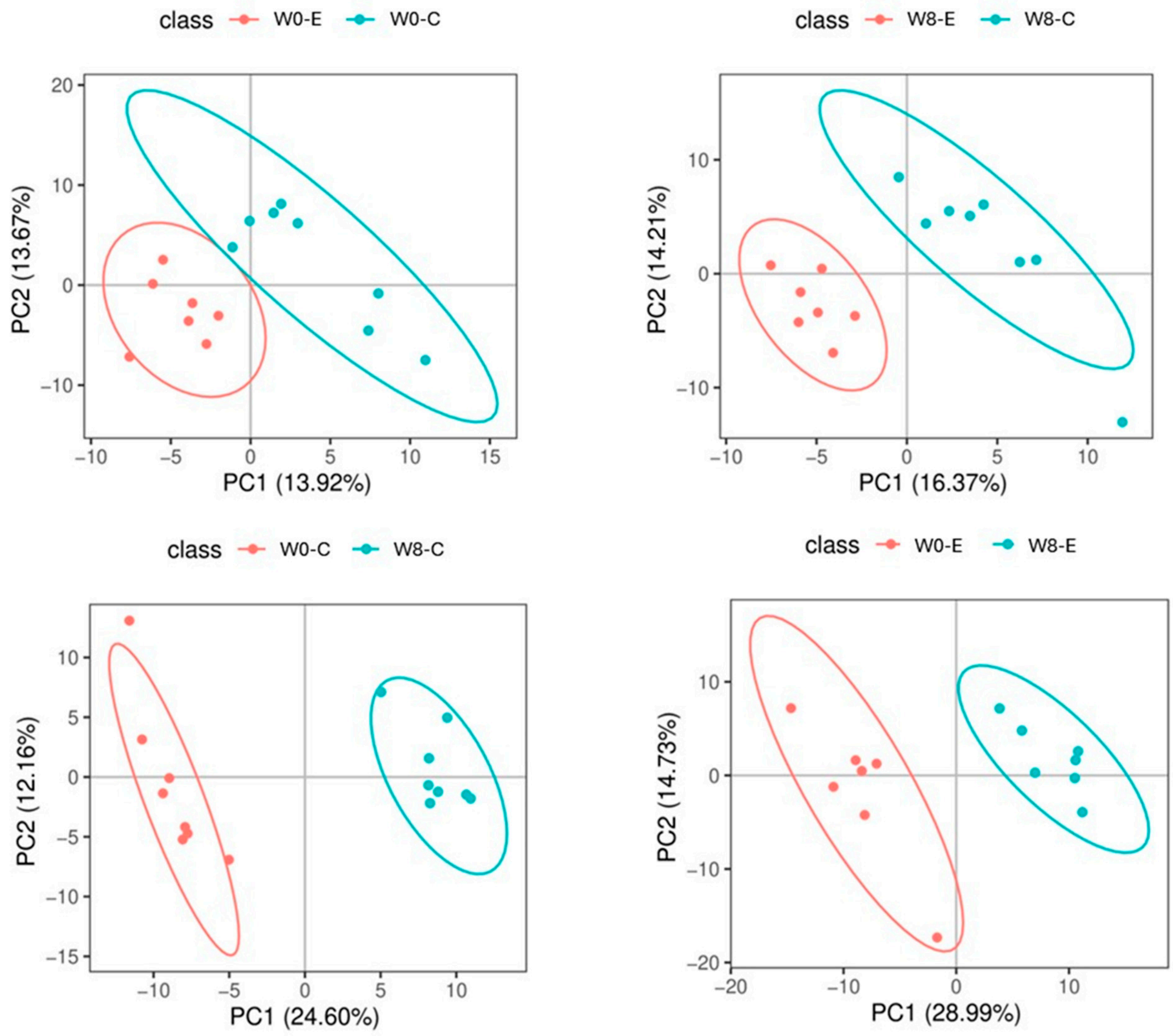

Supplementary Figure S1. Score graph of the PLS-DA analysis model of plasma-targeted metabolomic analysis

Horizontal coordinate: X-variate1 refers to principal component 1, and the number in parentheses represents the interpretation rate of X-variate1; Vertical coordinate: X-variate2 refers to principal component 2, and the numbers in parentheses represent the interpretation rate of X-variate2; Each point represents a sample, different colors represent different sample groups, and ellipses represent a 95% confidence interval.
